# Fatigue and recovery in ballet: Exploring the experiences of professional South African ballet dancers

**DOI:** 10.1186/s13102-024-01026-w

**Published:** 2024-12-03

**Authors:** Lania-Schane Smith, Quinette Abigail Louw, Yolandi Brink

**Affiliations:** Division of Physiotherapy, Department of Health and Rehabilitations Sciences, Stellenbosch University, Francie Van Zyl Drive, Parow Valley, Cape Town, 7500 South Africa

**Keywords:** Professional ballet dancers, Fatigue, Recovery, South Africa

## Abstract

**Background:**

Professional South African ballet dancers face a higher risk of overuse injuries and overtraining syndrome as compared to dancers from other countries; especially towards the end of a ballet season. Fatigue is a major factor leading to overtraining syndrome and injuries in professional ballet dancers. The study aimed to explore the fatigue and recovery experiences of South African ballet dancers, highlighting how they navigate these aspects and the strategies they employ for recovery.

**Methods:**

Snowball sampling was employed to recruit ten professional South African ballet dancers. Between March and June 2023, individual semi-structured interviews were conducted online using Microsoft Teams. The interview schedule, informed by previous research, delved into the dancers’ fatigue and recovery experiences. Interviews were recorded, transcribed, and analysed using inductive thematic analysis.

**Results:**

Seven main themes were developed from the data. Participants distinguished between mental and physical fatigue, highlighting the impact of mental fatigue on their well-being. They identified factors contributing to fatigue, including high external pressure, motivated behaviours, and expectations from audiences and management. These contributors often resulted in injuries, mandatory breaks, and sub-optimal performance. Participants accepted fatigue as inherent to their profession, emphasising mental resilience. Recovery practices included psychological, nutritional, and active strategies. The COVID-19 lockdown provided a respite from external pressures, allowing for improved recovery, but participants faced challenges upon returning to work. Gradually increasing practice time and exposure to external pressures assisted ballet dancers in effectively managing fatigue and enhancing their recovery as they returned to work.

**Conclusion:**

This study offers a perspective of fatigue and recovery in a sample of professional ballet dancers in South Africa. It emphasises the ongoing significance of fatigue and recovery and the necessity for effective strategies by arts organisations, mental health professionals, and academic researchers to support artists. The insights gained from this research could be crucial in redesigning training programs for dancers, adjusting training intensity and volume to enhance performance, and promoting recovery. Furthermore, this information serves as a strong rationale for adopting a holistic approach to fatigue and recovery, emphasising the importance of a collaborative effort to address these aspects effectively.

**Supplementary Information:**

The online version contains supplementary material available at 10.1186/s13102-024-01026-w.

## Background

Ballet dancers are a special combination of artists and athletes; performing artistic routines that require both physical skills and strength [1]. As time has passed, ballet has become more competitive and demanding, emphasising the importance of both technical and artistic abilities, especially in contemporary professional dance performances [2–6]. The demanding repertoire of ballet companies, which combines classical and modern works, intensifies the physical challenges faced by ballet dancers, contributing to the high incidence of musculoskeletal injuries in this profession [2; 7–10]. There is consensus globally and in South Africa that most ballet injuries are due to overuse, affecting mostly the foot and ankle [9; 11–17]. South African ballet dancers are at high risk for overuse injuries and the development of overtraining syndrome (OTS), particularly toward the end of a ballet season [18]. These injuries can be attributed to various factors, with fatigue being a well-documented risk factor for ballet-related injuries [19–22].

Fatigue is described in the literature as a “psychophysiological” condition that manifests as a decline in performance and is related to a real or perceived rise in the difficulty of a task, and/or the inability of muscles to maintain a certain level of strength during movements [23–25]. High quality performance depends on the ability of a dancer to produce and then sustain high levels of physical, technical, decision-making, and psychological skills [23]. A decline in any of these abilities may result from fatigue [23]. Fatigue is not only caused by physical factors but can have different origins such as sleep deprivation, gender (the subjective perception of fatigue is more common in women than in men), and psychological disturbances [25; 26]. These factors are known as “stressors” [27; 28]. Managing various stressors and fatigue is important to ensure positive adaptation to training, to ensure that the dancer is ready for a performance or competition and to reduce the susceptibility to injury and illness [29–32]. A recovery period and the use of recovery strategies are essential to achieve a balanced homeostatic state in the dancer and for the dancer to perform optimally [33; 34].

Recovery is a comprehensive process that addresses the rejuvenation of a dancer’s psychological, physiological, emotional, and social facets. This process involves the reduction, alteration, or elimination of stress to ultimately enhance the mental and physical condition of the individual [27; 29; 30; 35; 36]. A recovery-stress imbalance occurs when recovery strategies implemented by the dancers are insufficient and does not aid sufficiently in the restoration of homeostasis, which involves maintaining stability in bodily processes during times of increased pressure [28].

Fatigue can significantly affect both dancers and dance companies. For dancers, persistent fatigue can reduce performance quality, which may influence their roles and castings. Fatigue can also increase the risk of injury [19–22; 37]. For dance companies, the effects of fatigue are felt when dancers are unable to perform at their best or require extended recovery periods due to injuries, leading to disruptions in rehearsals and performances, and impacting the continuity of the production [12; 18; 23]. A better understanding of how professional South African ballet dancers experience fatigue and recovery is important as research has shown that injury prevention and performance enhancement strategies will assist with preventing the development of OTS and overuse injuries [18]. The aim of the study was to explore (1) how professional classical ballet dancers understand and experience fatigue and recovery, (2) which factors contribute to the experienced fatigue, and (3) which recovery strategies are implemented to reduce the fatigue experienced.

## Methods

### Research design

The goal of the research was to understand how individuals construct reality within their natural contexts [38]. The research paradigm was rooted in constructivism. Constructivism means that multiple interpretations of reality can be studied objectively and that humans construct knowledge through their experiences and interaction with the world [38; 39]. This study employed an exploratory descriptive qualitative (EDQ) study design to collect data via online, semi-structured interviews that took place between March 2023 and June 2023.

This study explored fatigue and recovery experiences among South African ballet dancers as it is a topic that has received little attention in the past [40]. EDQ methodology was used to investigate these experiences, providing insights from the dancers’ perspectives rather than merely documenting incidents [40–42].

### Setting

The research included two professional ballet dance companies in South Africa with a rich history within the arts and the development of the arts in South Africa. At the time, these were the only two professional ballet dance companies in South Africa. These South African dance companies are distinguished by their diverse repertoires, which include traditional full-length productions of major classical works, shorter ballets, original compositions, and a rich variety of dance styles. The dance companies employ between 20 and 30 full-time dancers, ranked according to their experience and skill sets. The highest rank is a Principal, followed by the Soloists and then the Corps de Ballet and apprentices. Ranks can further be divided into senior and junior roles within the rank with the seniors having more experience than the juniors. Because the companies are small, dancers must often fulfil more than one role, e.g., although they are employed as a Corps de Ballet, they might perform in Soloist roles. The staff members of dance companies are highly individualised and vary between the companies, with each company governed by a board of directors and managed by a company Chief Executive Officer.

A season is divided into a rehearsal phase and a performance phase. Pre-COVID-19, a performance phase could last from two weeks with ten performances, up to four weeks with approximately 24 performances. Post-COVID-19, for the two participating dance companies, a performance phase lasts about two weeks with eight to nine performances for the one company, and two to three weeks with 13 performances for the other. The rehearsal period of a season lasts for approximately two months. After a season, dancers receive one week holiday to rest and recover. Both companies are committed to the development of South African dancers and choreographers by creating opportunities for young talent, specifically from previously disadvantaged communities.

### Participant recruitment and sampling

Participants aged 19 years to 35 years were purposively recruited for this research study via the snowball sampling method [40]. Purposive sampling allows the researcher to choose participants to ensure the inclusion of a variety of participants [40; 43] and snowball sampling is used when potential participants are not easily accessible [44; 45].

The eligibility criteria required participants (1) to be employed by one of the two professional ballet companies; (2) to either be injury-free or have had experienced current or previous injuries; and (3) to be fluent in the languages spoken by the primary investigator (PI) (LS) to ensure effective communication during the interviews. English served as the official business language for the ballet companies, and all participants were proficient in English, with no participants expressing a need to be interviewed in another language (no participants were excluded based on language). Dancers who did not consent to participate were excluded from the study.

### Instrumentation

Participants’ demographic data was gathered through the utilisation of a demographic questionnaire developed by the authors. Gender/identity, age, rank in the company, dance experience (in years), and the number of injuries in the past year were collected for the purpose of describing the sample (Supplementary file 1).

The interview guide was developed by the authors, drawing on prior research in the field to ensure that the interview questions were context appropriate to lead to meaningful responses on the understanding and experiences of fatigue and recovery [46–49]. The interview guide included open-ended questions, based on the focus of the study (Supplementary file 2). The authors drew on previous research by Noh et al. [46] regarding the causes of occupational stress and coping strategy use among elite Korean ballet dancers, the study on the experiences of fatigue in patients with rheumatoid arthritis by Tack [49], and the research on factors influencing fatigue in Canadian university swimmers by Kennedy et al. [48], which informed and shaped the development of the semi-structured interview guide.

A pilot study was conducted with a semi-professional ballerina and a retired professional contemporary dancer to test the interview guide and the demographic questionnaire. Feedback from these participants helped refine the tools and ensure that the questions were clear and relevant to the dancers’ experiences. In particular, participant feedback from the pilot study and the first interview helped to ensure that the questions were relevant to the study’s focus on the understanding and experience of fatigue and recovery. Minor adjustments were made e.g., prompting questions concerning the impact of COVID-19 were adapted to ensure that study participants understood the focus was on how the pandemic and lockdowns influenced their experiences of fatigue and recovery, rather than on the impact of the illness itself. The feasibility of the recording method and the logistical aspects of the interview process were also assessed. By ensuring that the interview questions were grounded in relevant literature and refined through participant feedback, the study aimed to enhance the credibility of the findings, ensuring they genuinely reflect the dancers’ experiences [50].

### Ethical considerations

*Approval by regulatory authorities*: Ethical approval for conducting this study was obtained from the Health Research Ethics Committee at the Stellenbosch University. This study was conducted according to the Declaration of Helsinki and the Department of Health Guidelines for Good Clinical Practice (S21/10/208).

Participants in this low-risk study were informed about potential emotional experiences during interviews where they shared personal information, aimed at understanding causes and recovery strategies for fatigue among dancers. Electronic consent (e-consent) was obtained from all participants, following established steps to ensure understanding and compliance, with additional verbal consent for recording interviews [51; 52].

### Data collection methods

Two representatives (seeds) were contacted to aid in participant recruitment from the two dance companies. Study information was disseminated by these seeds to potential participants. For both companies, the timeframe between initial contact and participant recruitment was one and three days respectively. To protect the participants’ identities, participants were given unique codes such as P1 (participant one) which were used when referring to each participant. Participant one (P1) and participant six (P6), who belong to different dance companies and are acquaintances of the respective two seeds, contacted the PI and expressed their interest to participate. The scheduling of interview dates and times was coordinated through WhatsApp; this also allowed participants to ask questions before the interviews [53].

The research topic was introduced visually, either via a short PowerPoint presentation during the interview or a PDF document that was shared before the online interview, based on participant preference. Following their interviews, both P1 and P6 were encouraged to recruit additional participants, resulting in each recruiting four more participants. The data collection procedure was repeated for each consecutive participant. The PI conducted individual semi-structured online interviews (with video) which is an acceptable alternative to the face-to-face interview [54]. In this study, all the participants had adequate technical literacy to participate in the online interviews.

The PI kept a private research diary to take note of body language during interviews, as well as finer details such as facial expressions and tone of voice [55]. This assisted the PI with the interpretation of data. The research diary was also used to reflect on the research done. Regarding privacy limitations, the PI encouraged participants to do the interview in a quiet space with no distractions. The interviews lasted between 25 and 40 minutes.

### Data management and analysis

The data management process involved downloading the de-identified recordings. Interview transcripts were securely stored in a password-protected OneDrive folder on a dedicated computer. Thematic analysis, employing an inductive approach guided by Braun and Clarke’s method, was facilitated using the Computer Assisted Qualitative Data Analysis Software (CAQDAS), ATLAS.ti [56–59].

Phase one of the data analysis involved the researchers familiarising themselves with the data through simultaneous online interviews and transcription, aiding in identifying data saturation and initial patterns. Phase two saw the PI generating codes iteratively, initially coding each interview independently and then collaborating with co-authors to refine and categorise codes across all interviews [58]. Phase three focused on identifying emergent themes, with coding and theme development occurring concurrently to ensure comprehensive data exploration.

Phase four consisted of reviewing and organising themes, ensuring coherence and validity with continuous input from co-authors. Phase five involved defining and naming themes to reflect the essence of the data, a process that underwent rigorous verification and validation to enhance research reliability. Phase six culminated in producing a detailed report and code book, confirmed by co-authors, which highlighted seven overarching themes related to fatigue and recovery among South African ballet dancers. Throughout these phases, attention was paid to maintaining data integrity. This ensured that the research findings accurately reflected participants’ experiences and contribute to the broader understanding of fatigue management in professional dance contexts.

### Quality criteria

Trustworthiness of this study was ensured by the following criteria suggested by Lincoln and Guba [60; 61].

#### Credibility

Credibility was ensured through a well-established research method [62], data tools reviewed by co-authors to ensure comprehensiveness and alignment with research goals, and peer evaluation of analysed data [38]. Member checking of transcripts [38], structural coherence in reporting findings [62], and triangulation across interviews, questionnaires, and field notes were additional measures to enhance reliability [55]. Member checking was done after the interviews were transcribed. All participants checked their own transcript to ensure credibility of what was written.

#### Transferability

Thick description of the participants’ environment (e.g., institutional setting, resource availability, and social environment), as well as the research process, was done [38]. This helped the reader to assess whether the study findings were applicable to their context [38]. The socio-demographic questionnaires’ findings provided a descriptive profile of the participants, which added to the depth of the study findings [58].

#### Dependability and confirmability

Audit trails were maintained throughout the research process, documenting steps from proposal development through data collection and analysis to ensure replicability. The code-recode method was employed to ensure confirmability, with coding consistency checked on separate occasions [38]. Co-authors conducted peer review of findings for reliability of data.

#### Reflexivity

The PI, a physiotherapist, working in private practice in South Africa with an interest in dancer health and well-being, lead the research team. To mitigate bias, the PI maintained a research diary for self-reflection [38]. The PI was trained in qualitative methodology, interview skills, article writing, and the utilisation of the ATLAS.ti software.

## Results

### Socio-demographic information of participants

Ten professional ballet dancers who were employed by South African ballet companies in two different provinces of the country participated in the study. These were the only two provinces with professional ballet companies in the country. Inductive thematic analysis was used to determine data saturation [63; 64]. The median age was 26.4 years (range 19 to 35). The ranks of the participants were diverse and, collectively, the participants had 73 years of professional dance experience. The median years of experience were 7.3 years (range 1 to 17). The number of reported injuries in the past year were 16 for the group. Table 1 provides an overview of the socio-demographic characteristics of the participants.

**Table 1:** The participant demographics

Participant	Rank	Age	Gender	Professional experience (years)	Injury
1	Corps de Ballet	28	Female	9	3
2	Corps de Ballet (Senior)	27	Male	9	0
3	Apprentice (Senior)	24	Male	2	2
4	Corps de Ballet	26	Male	5	2
5	Apprentice	19	Female	1	1
6	Principal	35	Female	17	1
7	Corps de Ballet (Senior)	24	Female	6	1
8	Soloist (Senior)	30	Male	11	1
9	Soloist	28	Male	8	2
10	Corps de Ballet (Junior)	23	Female	5	3

The PI identified distinct patterns within the data. In alignment with the study’s objectives, these codes were organised into thematic categories. The thematic categories aimed at facilitating a comprehensive response to the research question. Seven overarching themes emerged regarding fatigue and recovery in professional South African ballet dancers. These themes were: (1) *Perception of fatigue*, (2) *Experience of fatigue*, (3) *Attitude towards fatigue*, (4) *Recovery practices*, (5) *Experience of recovery*,* (6) Perception of recovery*, and *(7) Attitude towards recovery.* No overlapping themes occurred (Tables 2 and 3).

**Table 2:** Themes and sub-themes for fatigue

Themes	Sub-theme	Sub-subtheme
**Perception of fatigue**	Mental	
	Physical	
**Experience of fatigue**	Contributors	High external pressure
		Ignoring tiredness
		Expectations from audiences and management
		Increased internal pressure
	Timing of fatigue	
	Consequences	Injuries
		Inability to work
		Impaired ability
**Attitude towards fatigue**	Acceptance	
	Mental resilience	

**Table 3:** Themes and sub-themes for recovery

Themes	Sub-theme	Sub-subtheme
**Recovery practices**	Psychological strategies	
	Nutrition and supplementation	
	Active strategies	
**Experience of recovery**	Facilitators to recovery	In-house medical professional
		Rank
	Barriers to recovery	High external pressure
		Pressure from company
**Perception of recovery**	Beneficial process	
**Attitude towards recovery**	Acknowledgement of importance of recovery	
	Acceptance of recovery not within control of the dancer	

#### Theme 1 – perception of fatigue

Participants’ perception of fatigue was illustrative of two forms of fatigue: mental fatigue and physical fatigue. Notably, a recurrent theme throughout their narratives was the interrelation between mental and physical fatigue, highlighting the interconnected nature of these phenomena.

#### Sub-theme – Mental fatigue

All participants clearly differentiated mental fatigue. Their perceptions of mental fatigue were not having the drive to complete an activity or task with full capacity.

*It’s one thing to have your body feel fatigue*,* but when you’re mentally fatigued as well*,* it’s hard to push through because you don’t have the support from your mind … I don’t have the capacity to push through.* (P5)

Most participants regarded mental fatigue as the most challenging form of fatigue due to its correlation with an increased perception of task difficulty among dancers.

*The mind is so important*,* and if the mind is fatigued*,* to me it’s worse than the physical side of it.* (P6)

P2 perceived her fatigue to be caused by heightened focus demanded, especially during performances, whereas five other participants (P4, P5, P6, P7, and P10) perceived their mental fatigue to be mostly due to elements such as the unwavering dedication inherent to their professional athlete roles. This underscores how the cognitive and emotional dimensions of their profession connect to shape their fatigue perception.

*It’s very disciplined and so sometimes the fatigue comes more from a mental state of having to be very disciplined and the consistency of training at a high level every single day.* (P7)

#### Sub-theme – physical fatigue

Participants described physical fatigue as a bodily occurrence, e.g., sluggish reaction times and weariness of muscles.

*Muscles don’t have that reaction when you needed to have it so that’s terrible.* (P1),

… *it can be like physical fatigue where your muscles are feeling tired or maybe from overwork or like when they’re very strained.* (P2)

Some participants also referred to other types of fatigue, such as spiritual, rehearsal and performance fatigue. However, these variants were not voiced consistently by all the participants but are still important as they describe a diverse perception of fatigue and illuminate the multifaceted nature of fatigue.

*Maybe also just like a spiritual fatigue*,* because you have a lot of self-belief and sometimes with a lot of performances you can almost question yourself and within rehearsals …* (P2).

### Theme 2 – experiences of fatigue

Three distinct sub-themes emerged within this theme. The first sub-theme delves into the contributing factors to fatigue that are catalysts for fatigue. The second sub-theme explores the timing of fatigue occurrence, and the third sub-theme explains the consequences of fatigue for the participants.

#### Sub-theme – contributors

The participants identified factors that contributed to the fatigue experienced.

##### High external pressure

External pressure refers to the physical demands placed on the dancer’s body from outside sources. External pressure affected all participants, leading to fatigue. Having to take on more roles in the company due to companies being small, little time off from work, performing repetitive movements in routines, as well as different dance styles all contributed to increased external pressure experienced by the participants.

*We’re a small company. Some of us do a lot.* (P8)

*Uh*,* so I think sometimes the workload can be quite a lot with very little time off.* (P1)

*Overdoing the exercise during class*,* repeating them a bit too many times.* (P9)

##### Ignoring tiredness

Participants mentioned that they often disregard the feeling of tiredness. Throughout the data two reasons surfaced that explained why the participants ignored this feeling. The first was their motivated behaviours. All the participants appeared to be very motivated and driven. This often led to behaviours such as ignoring tiredness and “pushing through”. Some participants admitted that their drive and motivated behaviours often resulted in fatigue or injuries that had detrimental effects on their careers.

… *for the first time I realised how important it was because my mind was pushing so much and (I was) so excited and strong*,* but the body was really taking a hit from it.* (P7)

*But for some reason in class*,* I was really trying to push strongly and really working hard*,* and I landed from a jump*,* I snapped my Achilles completely - full tear.* (P9)

The second reason was the influence of the “ballet culture”. One dancer offered a particularly insightful perspective describing the pervasive influence of the “ballet culture” that actively promotes the idea of pushing through.

*I think a lot of the older teachers and the training of ballet itself has this culture of pushing through injuries*,* like you’re strong and you don’t complain*,* and you push through. So*,* I think often you as a ballet dancer*,* especially a young dancer*,* ignore the way that your body is feeling I think I was feeling a bit tired*,* trying to ignore it*,* so I wasn’t noticing it so much in the body*,* even though if I had stopped*,* I would have noticed it a lot more.* (P7)

##### Expectations from audiences and management

Many participants agreed that the expectations set by company staff/management as well as audiences, contributed to the fatigue they experienced. A fear of facing criticism from audiences and the desire to please audiences, can be emotionally draining to the participants. Participants also mentioned that it often felt as if management disregarded their feelings of fatigue and pressure and in turn sets extremely high standards that dancers need to maintain. When company choreographers and directors set high artistic standards, dancers might feel pressured to excel, which could be emotionally exhausting. The constant emphasis on perfection and the feedback and criticism from company staff and management could increase the pressure experienced by dancers.

The pressure felt by certain participants holding a higher position or rank within the company was also identified as a notable element that contributed to fatigue. This is because higher performance expectations are set for them by choreographers and staff. They feel the pressure to excel and be perfect, in turn leading to more stress and a constant pressure to perform.

… *the management doesn’t really understand. They just see it as a weakness and not an actual issue.* (P1)

*You know the audience is there for the first time*,* so you have to be 100% every show.* (P8)

… *you know being a principal dancer is such a huge title to hold and it’s not stressful*,* but it’s a lot of pressure.* (P6)

##### Increased internal pressure

During the interviews, some participants shed light on an internal burden they experienced. This burden seemed to result from the profound demand for unwavering mental focus and attention while performing. The participants shared their struggles with self-doubt, particularly when it came to rehearsals and castings for performances, where the pressure to excel weighs heavily on their minds. Performance anxiety was also a notable aspect that contributed to the increased internal pressures experienced by these participants. Additionally, the fear of re-injury adds to the internal pressures and prevents some participants from excelling when performing.

… *you have to have a lot of self-belief and sometimes with a lot of performances you can almost question yourself and also with rehearsals and with casting.* (P2)

*I think the mental aspect*,* I mean obviously going on stage*,* you’ve got the nerves and the adrenaline*,* whereas you might not have that in the studio.* (P6)

*I felt a bit scared because of the injuries that (I) have had before. I feel quite scared when I’m fatigued.* (P10)

These quotes highlighted the complexity of the interaction between the mental state and the strive for excellence in performance, prompting the dancers to consider the possibility that mental state may indeed contribute to the experience of fatigue.

#### Sub-theme – timing of fatigue

Most participants agreed that they experienced the most fatigue during or just after a busy performance season. The participants attributed it to their busy schedules during performance seasons.

*I would say in December I was doing the Nutcracker and then I did the Nutcracker Cavalier and with the process doing lots of runs in the week.*(P2).

*So definitely after performance you’re fatigued.* (P6)

However, some participants also experienced fatigue during other times of the year. Four participants (P1, P5, P6, and P10) experienced increased fatigue mostly during busy rehearsal periods. One participant (P1) mentioned that back-to-back seasons caused the most fatigue, and one participant (P5) experienced the most fatigue at the end of a ballet year.

#### Sub-theme – consequences

It was clear that fatigue also held consequences for the participants. A recurring topic was the fact that participants were prone to injuries when fatigued. Another consequence that was frequently mentioned was the necessity for a “forced” or “mandatory” break because of injuries or exhaustion. Participants believed fatigue impairs their ability to dance, which is detrimental for their careers. A few participants also mentioned the development of physical illness, such as colds and flu, due to fatigue.

##### Injuries

Many participants suffered injuries because of fatigue, repetitive movements, ignoring illness and tiredness, and minimal pre-season training.

*You have to be extra careful because you can feel when you’re more fatigued*,* your injuries*,* your recurring stuff just appears more …* (P1).

*Yeah*,* really excited and everything. So*,* I was pushing myself*,* but I was still a bit sick and then I tore my ligaments.* (P7)

##### Inability to work

It became apparent that many participants must take a mandatory break from work due to injuries or exhaustion. They mentioned that this is detrimental for their careers.

*Six months before COVID I actually resigned*,* and I took a break for six months.* (P6)

*…actually*,* as I was doing jumps*,* little jumps*,* I developed a grade two tear of my Gastrocnemius on my right leg. So*,* I had to stop for a couple of weeks and then I started my rehab and I think in total it took me about 6–7 weeks to get back to my normal routine. Which was the longest I’ve ever been off for. Then I couldn’t do the performance. I think it happened like 2 ½ weeks before opening night. It was such a shame*. (P9)

##### Impaired ability

Some participants highlighted that when fatigued they were unable to perform at their best. They mentioned that fatigue impaired their full potential and that it feels like they could not do what they would have liked to do, despite being fully motivated.

… *if you’re feeling fatigued all the time and tired and you get frustrated because your body’s not doing what it’s supposed to do.* (P1)

*Well*,* it definitely inhibits your ability to do your job properly.* (P4)

### Theme 3 – attitude towards fatigue

#### Sub-theme - Acceptance

The participants displayed an attitude of acceptance towards their fatigue. They believed it is an inherent part of their chosen careers and that it is important to accept that as a professional dancer.

*So*,* the fatigue*,* it’s just part of the job.* (P6)

*I think the biggest thing is learning how to know YOUR body and so realising that you have chosen this profession*,* so you know the impact (it) is going to have: performing means late nights and less sleep*,* you have to learn to eat really properly more so than the average person.* (P5)

#### Sub-theme - Mental resilience

It was evident that most participants considered it crucial to have mental resilience as a professional ballet dancer. The participants displayed an attitude of acknowledging the important role mental resilience plays in managing fatigue. One participant even expressed that mental “fitness” is more important than physical “fitness” in the career of a professional dancer.

*Uhm*,* I think just like I realised*,* how important being mentally fit is*,* it’s almost more important to be mentally fit than physically fit.* (P5)

### Theme 4 - recovery practices

During the interviews the participants all mentioned recovery strategies they use to help prepare them (mentally and physically) for the intense training and performances. These recovery strategies could be grouped into three different categories, forming different sub-themes to this theme.

#### Sub-theme – psychological strategies

A recurring psychological strategy frequently mentioned during the interviews was the concept of “distraction”. This involved various techniques or activities aimed at diverting a dancer’s focus away from their dance and professional duties, ultimately leading to a state of complete relaxation and mental rejuvenation.

*I usually watch a movie to take my mind out of the normal daily life of things.* (P7)

*Distracting myself mentally.* (P9)

Other psychological strategies that were not reported on often, included Yoga (P2), meditation (P2), journalling (P5) and going on holiday (P6).

#### Sub-theme – Nutrition and Supplementation

Most of the participants reported that they use good nutrition and supplementation as a means of recovery. Participants felt that a nutritious meal helped them to recover from and experience less fatigue.

*I sometimes have low iron*,* so I make sure I eat red meat or pasta and mince and if I know I’ve got a hectic day*,* I’ll eat my pasta.* (P6)

*I always take vitamins and things like that. So*,* I’m on sort of quite set sort of different vitamins.* (P4)

#### Sub-theme – active strategies

The participants consistently reported the following active recovery strategies that they employed on a regular basis: Ice/cryotherapy and sleep.

*I’ve been icing my feet more recently that I really*,* really find helps me* (P5).

*Making sure you get enough sleep* (P1).

Stretching was another recovery strategy that the participants liked to implement daily. Participants mostly stretched after a long workday or just before bed at night, as this assisted them with relaxation and recovery.

*Stretching - that’s the norm*,* like evening and morning routine.* (P3)

### Theme 5 – experience of recovery

This theme has two sub-themes that explained how the participants experienced recovery in terms of the barriers and facilitators towards recovery.

#### Sub-theme – facilitators to recovery

##### In-house medical professional

Study participants saw the presence of an in-house medical professional (Physiotherapist) as a facilitator towards recovery. They believed the in-house medical professional helped them to identify weaknesses and reasons why they might be experiencing fatigue or injury. They also felt that the in-house medical professional helped by advocating for rest and load management when it was required.

*He’s the person who speaks to management. It’s not you having to walk like a little dog with your tail between your legs to say I’m so sorry*,* but I can’t dance. He manages everything and cc’s you in the e-mails.* (P1)

*He’s a physio at the studio. So*,* he helps in terms of*,* if you are finding yourself fatigued you can kind of figure out what is happening in the body and why it is happening …* (P2).

Only one of the two participating dance companies had an in-house medical professional who assisted with recovery.

##### Rank

The participants from one of the participating companies mentioned that their rank in the company could assist with recovery. The more senior you are, you only need to attend certain rehearsals and therefore have more time to rest.

*By the time you get to be a principal - I’m at that place where I pretty much only do the principal role and I might do a solo …* (P6).

#### Sub-theme – Barriers to recovery

Two factors emerged as barriers to recovery: high external pressure, and pressure from the company/management.

##### High external pressure

Most of the participants agreed that high external pressure acts as a barrier to recovery as participants do not have adequate time to rest. This leads to overwork and fatigue. High external pressure included aspects such as international tours, demanding seasons filled with numerous performances, and tightly packed schedules, especially when performance seasons are in full swing.

*In theory should be resting*,* but you can’t really rest because your job is completely physical. I mean*,* we don’t have days off because the work you miss kind of has to be done. It’s sort of like it’s not going to stop just for one person when there’s maybe 25 other people in the company.* (P4)

##### Pressure from company

Participants admitted that pressure from the company often prevents them from recovering fully. This is because they are not able to stop and rest as long as needed due to the companies being small, or general high standards within the company.

*There’s also that pressure to perform and because in the company there’s not like 500 dancers in the company and they can just chop and change. You do a lot more roles …* (P1).

*You can’t really say ‘Oh*,* my body’s feeling tired. I’m not going to do this today. So*,* you have to keep going and hope for the best.* (P10)

### Theme 6 – Perception of recovery

#### Subtheme - Beneficial process

Most of the participants viewed recovery as a beneficial process. They believed it increased motivation and caused a sense of positivity within. Some of the other benefits mentioned included heightened vitality and increased energy and drive. Participants knew that recovery and the recovery process was good for them, even though they did not always engage in these healthy behaviours.

*So*,* when you have recovered*,* it’s oh yes*,* I’m back I can do this. You’ve got energy. Feel excited.* (P1)

… *but I know once I’ve done my recovery process*,* then I feel much better. I feel stronger.* (P8)

Although two of the participants acknowledged that recovery is good and important; they also mentioned that the process of recovery and need for strict discipline felt like a repetitive process; however, they still implemented these strategies.

*Feel very much like Groundhog Day*,* like the same thing every day*,* but that’s also very good ‘cause then you do feel quite balanced.* (P2)

### Theme 7 – Attitude towards recovery

Two main sub-themes, related to the attitude towards recovery, emerged during the interview process. Participants portrayed similar attitudes towards recovery and the recovery process. The first sub-theme was an attitude of acknowledgement that recovery is essential for every athlete. The second was an attitude of resignation. Participants accepted that they are not always fully in control of their recovery and that external factors such as high external pressures caused poor recovery.

#### Subtheme - Acknowledgement of the importance of recovery

Participants were aware of and acknowledged the significance of allowing the mind and the body to recuperate after intense work/rehearsals or performances. Participants were proactive and had good intensions towards recovery and tried to be consistent with recovery practices to benefit their health. Whereas some participants implemented recovery strategies out of fear of re-injury after a traumatic injury.

*It stays quite consistent. I’ve over the years learned what works for my body.* (P6)

*Yeah*,* I’m quite consistent because I have a fear of reinjuring myself*,* so I’m quite consistent now.* (P10)

#### Subtheme - Acceptance of recovery not within control of the dancer

The participants mentioned that it often feels as if recovery is not manageable by them. This attitude emphasises the influence of external circumstances (e.g., fluctuating schedules during performance period), physical limitations, or other aspects (such as pressure from management) that could impact on a dancer’s ability to manage their recovery process effectively.

*It’s manageable in some ways and not in other ways. Like you can take care of yourself at home and at work*,* but there’s also that pressure to perform because the company is small.* (P1)

… *but that does become a bit random sometimes because if we’re in a show season*,* sometimes you’re finishing at 10 o’clock at night*,* getting home*,* then having to sort out food and then being in for work the next day at maybe 10*,* then sleep pattern goes out the window.* (P4)

In addition, the study further explored how the COVID-19 pandemic affected the fatigue and recovery experiences of the professional South African ballet dancers. This was in terms of the Level 5 lockdown that South Africans experienced. During this time, South Africans were not allowed to leave their homes. Participants were also not allowed to go to work or perform. The participants mentioned that they were following Zoom classes from home, which was not always ideal as they struggled to dance on tiles at home (P1). Regarding fatigue and recovery experiences, participants mentioned collectively that they saw the Level 5 lockdown as a positive, as they had time to rest (P1, P2, P4, P7, P8, P9). External pressures were much less, and they were not pressured to perform. This in turn lead to increased recovery and no fatigue being experienced (P10). Participants did, however, mention that after the lockdown, when they were allowed to go back to work, they experienced increased fatigue levels initially, some even experienced a few injuries due to the lack of training during the lockdown period (P1, P10). The participants mentioned that both companies managed the situation effectively by increasing the practice and external pressures gradually to assist participants with positive adaptation to training stresses (P1).

## Discussion

The aim of this study was to explore the experiences of fatigue and recovery in professional ballet dancers in South Africa, utilising online semi-structured interviews with ten participants. Seven overarching themes, indicating the multifaceted nature of fatigue and recovery, were identified that best describes the professional ballet dancers’ experiences of fatigue and recovery. According to our knowledge, no other study has previously reported on this topic within the professional ballet dance environment of South Africa.

### Exploring the experience of fatigue

Participants distinguished fatigue from ordinary tiredness, identifying both physical and mental forms, consistent with Magrath et al. [65], who found similar categorisations in undergraduate contemporary dancers. Magrath et al. [65] also found that undergraduate contemporary dancers experienced mental fatigue as a lack of concentration, motivation, and communication, reflecting our findings. Additionally, participants in our study emphasised the interconnectedness of mental and physical fatigue, which aligns with the observations of Van Cutsem et al. [66]. Their systematic review examined how mental fatigue affects the physical abilities of endurance athletes. They found that increased mental fatigue led to a heightened perception of effort and a decrease in both the duration and intensity of physical tasks. These findings also indicate similarities between the experiences of fatigue in our study and the newly proposed framework of fatigue by Behrens et al. [24].

The participants’ experiences can be categorised as *activity induced state fatigue* [24]. This concept entails an acute change in both motor and psychological performance, coupled with an increased perception of weariness induced by a cognitive or motor task [24]. Within this framework one can further distinguish between motor and cognitive fatigue, again mirroring the findings of the current research. Behrens et al. [24] also terms the increased perception of fatigue as the *perceived motor and cognitive fatigue* which depends on the mind-body state of the individual. In our research, it became evident that the perception of fatigue depended on the participants physiological and psychological state.

Our findings indicate that fatigue was influenced by several contributing factors, with external pressure emerging as the most prominent. Kellmann [67] noted that non-sport-related stress, combined with high training loads, can lead to fatigue and ultimately burnout. Participants in our study often ignored their fatigue, driven by strong motivation and a relentless work ethic. This aligns with research showing that highly motivated dancers are particularly vulnerable to fatigue and burnout due to their perfectionist tendencies [21; 68–70]. Magrath et al. [65] found that undergraduate contemporary dancers prioritised good grades over injury recovery. This study also showed that the “ballet culture” normalised pain and encouraged fatigue suppression [71]. While this may initially promote success, it ultimately harms health. Addressing these cultural and motivational factors is essential for fostering healthier practices and better support for dancers.

The experience of fatigue in this study varied by rank, aligning with findings by Walker and Nordin-Bates [72], who observed that ballet dancers of different ranks faced varying levels of pressure and distinct expectations. Similarly, De las Heras-Fernández et al. [73] reported lower anxiety scores in dancers not portraying lead roles. This study’s findings illuminate the internal dynamics within a ballet company and their impact on fatigue.

The emotional landscape of ballet plays a significant role in fatigue. Ballet is a form of communication between the dancer and the audience, leading to a range of emotional experiences [74]. Dancers often face negative emotions, such as fear of criticism and a desire to please the audience, contributing to their overall fatigue [74]. A study by Shikanai and Hachimura [74] found that dancers experience both positive and negative emotions during performances. The researchers noted that while dancers initially feel nervous due to the audience’s presence, they gradually enjoy the performance and focus on conveying the story. This information can help strengthen emotional support systems for dancers and contribute to their well-being and coping mechanisms in live performances. These support systems will assist with the high internal pressures often experienced by professional dancers [72].

In the current research study, a few other internal pressures also led to dancers’ overall development of fatigue which aligns with other research. Vassallo et al. [75] found that dancers often fear the impact of injuries on their employment and casting opportunities. This fear arises from the high-stakes nature of the dance industry, where injuries can lead to missed performances and potentially affect future casting decisions. Consequently, dancers may downplay injuries and push through pain, risking long-term health issues and highlighting the need for improved support and injury management in the profession. These contributing factors emphasise that the aetiology of fatigue is multifaceted, influenced by a combination of physical, psychological, and environmental factors.

Fatigue in this study predominantly occurred during times of heightened focus and demand. This finding correlates with the study by De Wet, et al. [18], who found that stress increased and recovery decreased during performance phases of the year in professional South African ballet dancers. Strahler and Luft [76] conducted a case study, collecting self-reported measures and biological markers throughout a dance season to determine acute and chronic stress in a professional ballroom dancer. Their results indicated that ballroom dance competitions are a significant biological stressor compared to regular training days, consistent with our findings of increased fatigue during the performance season [76].

In our study it is evident that injuries emerge as the primary outcome to fatigue; correlating with previous research [19–22]. Interviews underscored that fatigue notably impairs performance, affecting concentration and the ability to execute movements precisely. Research by Knicker et al. [23] in diverse sports corroborates that fatigue can impair critical skills such as technical proficiency and decision-making.

Many participants accepted that fatigue is an inherent part of their career. The participants did, however, acknowledge the importance of mental resilience within their field of work. According to the American Psychological Association, psychological resilience is the ability to adapt effectively when stress is experienced [77]. Previous research found that developing and having mental resilience is an integral part of being a professional athlete to acquire and maintain high levels of performance [77]. Without the necessary resilience, athletes (especially female athletes) may encounter unfavourable developmental outcomes, including strained relationships with coaches, negative peer influences, and difficulty in effectively reacting to adverse stimuli [77].

### Exploring the experience of recovery

Recovery practices are strategies used by dancers to help reduce the negative effects of stress [29]. The study by Grove et al. [29] showed that optimum recovery strategies varied from person to person as previously stated in the review done by Jeffreys [27]. This was also noticed in the current research study.

The recovery strategies most often used by participants in the current study included *supplementation*, *nutrition*, *active strategies* (cryotherapy, sleep and stretching), and *psychological strategies* (distraction, such as walking a dog or watching a movie). Previously, Magrath et al. [65] found that participants preferred sleep, rest, nutrition, hydration, and work-life balance as key recovery strategies; and Koutedakis [68] reported that counselling, sleep, saunas, massage, aromatherapy, and hydrotherapy were beneficial for dancers to help manage burnout. Progressive muscle relaxation was effective to improve sleep onset latency in elite ballet dancers who tend to be more anxious [36]. Jeffries et al. [78] suggested that modifying the training load distribution and including recovery days and short periods of reduced load were possible interventions to assist with recovery in professional ballet dancers. Therefore, it may be beneficial to consider integrating a combination of these recovery strategies into a structured regimen, along with adjusted training loads. This approach could help enhance dancer recovery and reduce the risk of burnout, ultimately supporting long-term performance and well-being.

Participants faced challenges maintaining consistent recovery strategy use, especially nearing or during performance phases of the season, particularly with sleep and nutrition management. De Wet et al. [18] recommended continuous monitoring of recovery-stress states, educating dancers on the importance of recovery and social support, and implementing practical recovery techniques especially during these demanding performance seasons.

Facilitators to recovery included participants’ rank and the presence of an in-house physiotherapist. Higher ranked dancers received more tailored training and faced fewer physical demands. Participants identified two main barriers to recovery which hindered consistent use or proper application of recovery strategies: management pressure and external demands which left little time for recovery. Similarly, Shell et al. [79] found that lack of time was the primary reason swimmers did not complete their recovery processes, suggesting that recovery should be integrated into training plans to improve compliance and effectiveness.

Participants accepted fatigue and they believed that, although recovery is important, it is not always within their control to consistently implement due to external factors. Magrath et al. [65] confirmed that undergraduate contemporary dance students often cannot rest due to management creating dancer schedules without incorporating input from the dancers themselves. This indicates that a collaborative approach in the professional dance environment between the dancers and management could be a possible way of improving and promoting recovery in this setting.

## Clinical implications

This study offers insights that can inform practical applications within the ballet community. Recognising the distinction between physical and mental fatigue, as well as their interrelatedness, is important for healthcare professionals to diagnose and manage fatigue-related conditions effectively within this specific population.

The findings of this research highlight the significant impact of external pressures, such as rigorous training and competitive environments, on dancers’ fatigue levels. This emphasises the need for stakeholders to implement better workload management practices and support systems that consider these external demands. Additionally, the study identifies effective recovery strategies, such as scheduled rest, tailored nutrition, and mental health support, which are essential for managing fatigue, particularly during peak performance periods.

## Limitations

This was the first study exploring the experiences of fatigue and recovery in professional ballet dancers; however, the study has limitations. Since the researchers used snowball sampling, sampling bias cannot be excluded. Individuals with certain characteristics more likely volunteered to participate in this research influencing the findings of the study.

Ten online interviews were conducted to gather data. Even though saturation was achieved, the sample size was still small which could mean limited representativeness of the group. Although the online data collection methods contributed to the strengths of the study, it also served as a limitation. Two participants struggled with connectivity and others did not have a private room where they could partake in the interview, hampering the privacy of data collection and causing unavoidable noise interference.

Online data collection also means that a limited trust relationship is built between researcher and participants. Reading the body language of the participant were more challenging during online interviews due to limited visibility of the participant. The study might be susceptible to recall bias as the participants were asked to remember occurrences from the past. Future research might incorporate longitudinal study designs where data is collected over an extended period.

Another limitation of the study was the lack of a deeper analysis into the types of injuries (such as overuse- and acute injuries) the participants had experienced over the past year (which may or may not have affected their abilities to perform well or at all). This omission hindered a more comprehensive understanding of how different injury types might impact the participants’ dancing careers and their performance over time. Future larger scale studies could also explore the relationship between demographic data collected in this study (professional experience, rank and injury) and preferred recovery strategies as this could be useful information in the development of tailored recovery strategies and injury prevention regimes.

## Conclusion

The aim of the research was to explore the experiences of fatigue and recovery within the professional ballet dance population in South Africa. Through a qualitative, exploratory descriptive research design, it was possible to delve deeper and describe the participants’ experiences. The main research findings were that the participants experienced fatigue as a psychophysiological manifestation of weariness that impacted the quality of their work (dance performance) and that often led to devastating, career impacting injuries and an inability to work. The research also confirmed that fatigue is caused by various aspects such as high internal and external pressures, ignoring tiredness and pressure from the company and audiences.

Participants most often experienced fatigue during a busy performance season, which could possibly be due to high stress experienced and poor adherence to recovery protocols. Participants do, however, portray an attitude of acceptance towards fatigue and believe that it is an inherent part of their chosen careers and, as professional athletes, they valued mental resilience. They deemed recovery important and had their own recovery routine. The participants did however mention that recovery is influenced by external factors and that it is not always within their control.

The current research adds value by offering insight into the experiences of professional South African ballet dancers. These insights can be used to inform training programmes in the companies, plan injury prevention strategies and ensure a holistic approach to fatigue and recovery in the professional ballet dance community.

## Electronic supplementary material

Below is the link to the electronic supplementary material.

Supplementary Material 1

## Data Availability

The data transcripts are not available publicly due to a signed agreement between the researchers and participants.

## Abbreviations

COVID-19: Coronavirus Disease 2019
OTS: Overtraining syndrome
EDQ: Exploratory descriptive qualitative
CAQDAS: Computer assisted qualitative data analysis software
e-consent: Electronic consent
e-mail: Electronic mail
PI: Primary Investigator
LS: Lania Smith
