# Supplementary material for: Fatigue and recovery in ballet: Exploring the experiences of professional South African ballet dancers
Source: BMC Sports Sci Med Rehabil. 2024 Dec 3;16:237. doi: 10.1186/s13102-024-01026-w (PMC11616340; doi:10.1186/s13102-024-01026-w)
Supplement: Supplementary file 1 — Supplementary Material 1 [file 13102_2024_1026_MOESM1_ESM.pdf]

Supplementary file 1: Demographic questionnaire

Gender	
Age	
Rank in Ballet company	
Years danced/Experience	
Number of injuries in the past year	

Supplementary file 2: Interview schedule

Purpose:

- The Purpose of this interview is to explore professional South African ballet dancers' experiences of fatigue, as well as their experiences of recovery.
- I would like to explore your understanding of fatigue as well as your personal experiences.
- I would like to explore the contributing factors to the fatigue that you experience.
- I'm interested in discovering what recovery strategies you use to reduce the amount of fatigue you experience, whether there are any facilitators or barriers to recovery strategy use and how recovery strategies influence the fatigue you experience.

Ethics:

- All the data is being recorded, as mentioned in the informed consent form that you already signed.
- You have the right to stop the interview at any time, leave the interview at any time and request the recording be deleted at any time in the session.
- Data is only being used for research purposes and is confidential.

Please bear in mind that the questions asked, refer to your understanding and personal experiences of fatigue. Please try and answer to the best of your abilities. This research might benefit future dancers because it will inform and educate dancers with regards to fatigue and recovery which will help ensure optimal performance of dancers. The study findings will also assist company staff and medical staff with injury prevention programme development and the development of performance enhancement programmes. As this is an explorative study, it will give direction and a sound base for future researchers to work from. Future researchers will be able to use

this research to develop interventions that could assist dancers even further. Any questions at this point?

Interview schedule:

**General opening questions**	
**Question One**	How long have you been dancing for?
**Question Two**	Why did you choose Ballet as a career?
**Questions about fatigue experiences**	
**Question Three**	How do you understand or interpret the word fatigue? How would you describe fatigue?
**Question Four**	Can you describe an experience or an instance where you experienced fatigue? How did it make you feel?
**Question Five**	What do you think caused the fatigue you experience?
**Question Six**	Have you ever had an injury before (overuse or traumatic)? If so, which area of your body and during what time/season of the ballet year was it?
**Question Seven**	What do you think contributed to the or caused the injury?
**Question Eight**	Have you ever been formally diagnosed with chronic fatigue or Overtraining syndrome by a medical professional?
**Questions about recovery**	
**Question Nine**	How do you deal with the fatigue you experience?
**Question Ten (Probing question)**	What recovery strategies do you use?
**Question Eleven**	What prevents you from recovering fully?
**Question Twelve**	Are there things in your current day or schedule that contribute to/ or assists with your recovery?
**Question Thirteen**	How do recovery strategies influence/affect the fatigue you experience? How does it make you feel?
**Questions about the COVID-19 pandemic and fatigue/Recovery**	
**Question Fourteen**	How did the/ does the COVID-19 pandemic impact your experience of fatigue?
**Question Fifteen**	How did the COVID-19 pandemic influence your recovery?
